# Assessing the Impact of Older Americans Act–Funded Services on Caregiver Stress

**DOI:** 10.1093/geroni/igaa057.1145

**Published:** 2020-12-16

**Authors:** Racheal Chubb, Janet Wilmoth

**Affiliations:** 1 Syracuse University, Felts Mills, New York, United States; 2 Syracuse University, Syracuse, New York, United States

## Abstract

Funding for OAA programs that support caregivers and care recipients has steeply declined since 2010. This is potentially problematic given these programs provide services that may reduce caregiver stress. To better understand what and for whom caregiving services reduces stress, we used data from the National Survey of Older Americans Act Participants (NSOAAP) to estimate logistic regression models predicting caregiver stress. These models include the following measures: services provided to the care recipient, services received by the caregiver, and caregiver satisfaction with services received; caregiver health, age, race, and gender; and care recipient health, age, gender, relationship to the caregiver, and coresidence with the caregiver. The results indicate that caregivers who were satisfied with services were less likely to be stressed compared to those who are not satisfied with services. Those who received respite care and counseling services were less likely to be stressed than those who attended classes and training. Consistent with the literature, caregivers who reported better health or cared for someone in better health were less likely to be stressed. Caregivers were also more likely to be stressed if they provided help with medical care compared to ADLs or if they lived with the care recipient. Overall, the results underscore the importance of continued, and possibly expanded, OAA funding for caregiver support services, especially those that provide respite and counseling to individuals who are providing care to frail and co-residential older adults.

